# Scientific evidence based rare disease research discovery with research funding data in knowledge graph

**DOI:** 10.1186/s13023-021-02120-9

**Published:** 2021-11-18

**Authors:** Qian Zhu, Ðắc-Trung Nguyễn, Timothy Sheils, Gioconda Alyea, Eric Sid, Yanji Xu, James Dickens, Ewy A. Mathé, Anne Pariser

**Affiliations:** 1 Division of Pre-Clinical Innovation, National Center for Advancing Translational Sciences (NCATS), National Institutes of Health (NIH), Rockville, MD 20850 USA; 2 ICF International Inc, Rockville, MD USA; 3 Office of Rare Diseases Research, National Center for Advancing Translational Sciences (NCATS), National Institutes of Health (NIH), Bethesda, MD 20892 USA

## Abstract

**Background:**

Limited knowledge and unclear underlying biology of many rare diseases pose significant challenges to patients, clinicians, and scientists. To address these challenges, there is an urgent need to inspire and encourage scientists to propose and pursue innovative research studies that aim to uncover the genetic and molecular causes of more rare diseases and ultimately to identify effective therapeutic solutions. A clear understanding of current research efforts, knowledge/research gaps, and funding patterns as scientific evidence is crucial to systematically accelerate the pace of research discovery in rare diseases, which is an overarching goal of this study.

**Methods:**

To semantically represent NIH funding data for rare diseases and advance its use of effectively promoting rare disease research, we identified NIH funded projects for rare diseases by mapping GARD diseases to the project based on project titles; subsequently we presented and managed those identified projects in a knowledge graph using Neo4j software, hosted at NCATS, based on a pre-defined data model that captures semantics among the data. With this developed knowledge graph, we were able to perform several case studies to demonstrate scientific evidence generation for supporting rare disease research discovery.

**Results:**

Of 5001 rare diseases belonging to 32 distinct disease categories, we identified 1294 diseases that are mapped to 45,647 distinct, NIH-funded projects obtained from the NIH ExPORTER by implementing semantic annotation of project titles. To capture semantic relationships presenting amongst mapped research funding data, we defined a data model comprised of seven primary classes and corresponding object and data properties. A Neo4j knowledge graph based on this predefined data model has been developed, and we performed multiple case studies over this knowledge graph to demonstrate its use in directing and promoting rare disease research.

**Conclusion:**

We developed an integrative knowledge graph with rare disease funding data and demonstrated its use as a source from where we can effectively identify and generate scientific evidence to support rare disease research. With the success of this preliminary study, we plan to implement advanced computational approaches for analyzing more funding related data, e.g., project abstracts and PubMed article abstracts, and linking to other types of biomedical data to perform more sophisticated research gap analysis and identify opportunities for future research in rare diseases.

**Supplementary Information:**

The online version contains supplementary material available at 10.1186/s13023-021-02120-9.

## Background

A rare disease is defined as any disease that affects fewer than 200,000 individuals in the United States. There are an estimated 25–30 million Americans that are affected by one of approximately 7000 different rare diseases, most of which are poorly understood with unclear underlying biological mechanisms. This knowledge gap leads to challenges for patients, clinicians, and investigators. Patients affected by a rare disease experience delays in diagnosis, as well as a lack of available treatments, clinicians often have limited clinical knowledge and experience impedes their clinical decision making, and investigators struggle with limited patient data and sparse funding for research across most rare diseases [1]. To help address these challenges, we proposed a detailed analysis of research funding data to (1) enhance understanding of the current funding situation and potential funding opportunities in rare diseases, and (2) identify gaps among current research activities in rare diseases that may be primed for new research.

Compared to common diseases that are more highly prevalent in the population, such as depression or heart disease, research funding is often scarce for rare diseases, in part due to the relatively small number of people affected, and lower prioritization of funding based on the perceived burden of the disease [2]. In fiscal year 2019, the National Institutes of Health (NIH) were appropriated $39 billion [3], of which only $38 million (0.1%) was awarded to study a wide range of rare diseases [4]. Similarly, in the UK, less than 1% of the annual funding budget from three out of the top four UK funders were directed towards rare disease research [5]. A lack of funding results in less research aiming to understand disease etiology, identify biomarkers for disease diagnosis, develop of novel medications and associated clinical trials, and ultimately, absent treatment options. To solicit and attract more funding for innovative research in rare diseases, there is an urgent need to better understand and assess the current funding situation/trend and address gaps found to persist from retrospectively tracing funding history.

To review the funding landscape, Stehr et al. examined funding circumstances for Batten Disease, a group of rare nervous system disorders, by extracting funding information from publications. Interestingly they discovered 193 funding agencies had supported Batten Disease research to date, which might encourage researchers to continue their pursuits and expand their studies, moving key findings from discovery to application phases [6]. Franceschi et al. led a study to characterize recent NIH funding for diagnostic radiology departments at US medical schools [7]. To inform decisions on research direction, Ma et al. examined over 43,000 scientific projects funded over the past three decades and established collaboration networks that revealed major ramifications on future research strategy and government policy [8]. Packalen et al. performed an analysis on a comprehensive corpus of published biomedical research articles, and found that edge science framed with novel basic science ideas is more often funded by the NIH than less novel science [9]. These studies either limited their review of the funding landscape to a few specific diseases or diagnostic protocols, or assessed research direction globally. In our study, we aimed to develop an approach to systematically overview funding trend across different disease categories or individual diseases, and identify research gaps globally or locally (i.e., for individual disease category or disease) to further support rare disease research.

To overcome the challenge of handling large amounts of data accumulated given scientific advancements, knowledge graphs have attracted a lot of interest in the biomedical domain, as they can be leveraged to semantically represent relationships among large-scale data [10]. Ding, et al. constructed a PubMed knowledge graph (PKG) by extracting bio-entities from 29 million PubMed abstracts with integrated funding data through the NIH ExPORTER, in order to measure scholarly impact, knowledge usage and transfer, and profile authors and organizations based on their connections with bio-entities [11]. In our previous work, we developed an integrative knowledge graph in Neo4j [12], named NCATS GARD Knowledge Graph (NGKG) that contained a large volume of biomedical and research data pertinent to rare diseases [13]. Inspired by these published studies, we proposed to aggregate and represent funding data for rare diseases in a semantic manner as a knowledge graph. While the PKG incorporates publications and funding to gain insights and approaches to connect researchers with common research interests, our primary goal is to not only assess the funding landscape of rare diseases, but also identify research status and knowledge gaps, which can be applied to promote novel research for rare diseases.

## Materials

### Rare disease resources

In this study, we incorporated rare disease information from the Genetic and Rare Diseases (GARD) [14] and Monarch Disease Ontology (MONDO) [15]. GARD is a public health information center managed by the Office of Rare Diseases Research (ORDR) within the National Center for Advancing Translational Sciences (NCATS), and retains curated disease information for about 7000 rare diseases. MONDO is a semi-automatically constructed ontology that merges multiple disease resources to yield a single coherent merged ontology [15]. We accessed GARD and MONDO from our previously developed NCATS GARD Knowledge Graph (NGKG) in Neo4j [13, 16].

### NIH funding resource

Research Portfolio Online Reporting Tools Expenditures and Results (RePORTER) is a key component of the NIH’s “open government” initiative to provide more transparency into NIH activities, including information on NIH expenditures and the results of NIH supported research [17]. ExPORTER provides bulk administrative data found in RePORTER to the public for detailed analyses or to load into their own data systems [18]. ExPORTER provides downloadable versions of the data accessed through the RePORTER interface, and includes information about projects, publications, patents, and clinical studies. In this study, we downloaded and cleaned the funded projects and associated publications from ExPORTER, and stored the data in a MySQL database, from where we obtained data for the analysis described in this study.

## Methods

### Rare disease data preparation

We extracted 6305 GARD rare diseases from the NGKG in Neo4j [13, 16]. In order to adopt disease categories from MONDO to organize these extracted rare diseases, we only included 5001 GARD rare diseases that have one-to-one exact mapping to MONDO based on one MONDO property, “MONDO:equivalentTo”. As a proof-of-concept with minimized manual validation, we did not include one-to-many, many-to-many or any other partial mappings between GARD and MONDO concepts, which will be included in a future study.

To enable review and analysis of research funding by disease categories, we mapped GARD diseases to MONDO disease categories. MONDO contains three main branches in its disease classification tree, namely, “Disease Characteristic”, “Disease or Disorder” and “Disease Susceptibility”. In this study, we focused on the branch of “Disease or Disorder” (MONDO: 0000001) in MONDO obo file [19], from where we extracted 32 root disease categories, including congenital abnormality, acute disease, disorder involving pain, serpinopathy, psychiatric disorder, visceral myopathy, and post-infectious disorder, etc. (A complete list of 32 root disease categories can be found in Additional file 1) We mapped those 5001 GARD diseases to the 32 root categories accordingly by iteratively searching the MONDO disease hieratical tree. It is worthy to note that most GARD diseases map to more than one MONDO disease categories.

### NIH funded project identification for rare diseases

NIH ExPORTER provides detailed information about funded projects, including project titles and project abstracts in free text. We assumed that if the disease name is mentioned in the project title, it is likely that the project was proposed to conduct research investigation on this disease. Hence, we proposed to map GARD disease names to project titles, to identify a list of funded projects for each individual GARD disease via two steps as described below. Before mapping, we excluded projects with invalid project titles, such as, “13.358”, “CFDA NO. 13-299”.

#### Mapping based on name match

We executed ‘LIKE’ SQL operator as exact string match to identify projects with disease names mentioned in the project titles from the MySQL database, which stores the cleaned funding data. To avoid any mis-mappings, we applied not only full disease names but also alternative disease names in the ‘LIKE’ operator. Since abbreviations occurring in disease names and/or synonyms might cause incorrect mappings, disease names and synonyms with less than 4 characters were excluded for mapping.

#### Mapping based on semantic annotation.

**Fig. 1:**
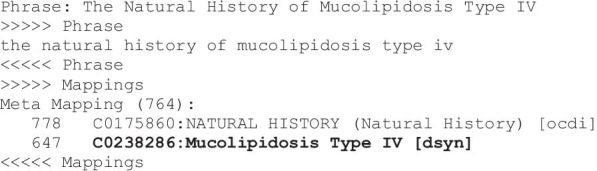
Annotation results generated by MetaMap (The fields in bold were extracted and applied to map to GARD diseases, “C0238386” is UMLS identifier, “Mucolipidosis Type IV” is UMLS preferred name, “[dsyn]” is one UMLS semantic type, “Disease or Syndrome”)

As a complementary step, we semantically annotated project titles in free text by using MetaMap annotator [20], and then mapped GARD diseases to those generated annotations. MetaMap annotator produces a list of annotations in the Unified Medical Language System (UMLS), including UMLS semantic types, its preferred names, etc. Figure 1 shows a snapshot of annotation results generated for a project entitled “The Natural History of Mucolipidosis Type IV”. To avoid any incorrect mappings, we excluded those annotated concepts with less than 4 characters for further analysis, for example: “RNS (UMLS: C1850106)” as one annotation for the project title of “HAMPTON INSTITUTE'S CONTINUING EDUCATION PROGRAM FOR RNS”.

In order to establish the mappings between funded projects and GARD diseases, we mapped annotations generated from each project title to GARD diseases based on UMLS mappings since MetaMap output is in the UMLS, as shown in Fig. 1. We retrieved mappings between GARD diseases and the UMLS via two steps. First, we obtained the UMLS mappings that were curated by GARD. Next, we obtained the UMLS mapping from MONDO for the GARD disease if the GARD disease concept was exactly matched to the MONDO concept and this MONDO concept had an external mapping to the UMLS. For instance, Bloom syndrome (GARD:0000915) is exactly matched to one MONDO concept of Bloom syndrome (MONDO:0008876) that is mapped to one UMLS concept (UMLS:C0005859). Thus, with UMLS:C0005859, we were able to map this GARD disease to one project entitled “BLOOM'S SYNDROME--DNA LIGASE AND IMMUNODEFICIENCY” with one annotation of Bloom Syndrome (UMLS:C0005859).

### Data model

Once we established the connections between GARD diseases and funded projects through the above steps, we designed a data model to semantically capture and represent different types of data extracted from those funded projects and their associated data, such as, publications or principal investigators.

#### Primary class definition

We defined seven primary classes, namely, Disease Category, Disease, Funded Project, Funding Agent, Principal Investigator, Publication, and Journal. These classes capture a full spectrum of information present in NIH funding data and enable linkages to other different types of data for directing more sophisticated research on rare diseases, which will be described in the “Discussion” section.

#### Object property definition

To capture semantic relationships among those primary classes, we defined object properties as shown in Table 1.

**Table 1:** Defined object properties

Object properties	Subject classes	Object classes
isClassOf	Disease	Disease category
isInvestigatedBy	Disease	Funded project
isFundedBy	Funded project	Funding agent
hasPublication	Funded project	Publication
hasFundedProjectOf	Principle investigator	Funded project
isPublishedOn	Publication	Journal

#### Data property definition

We defined a list of data properties shown in Table 2 to link data values for each individual concept.

**Table 2:** Defined data properties

Data properties	Corresponding classes
category_id^a^, category name	Disease category
gard_id, isRare, mondo_id, disease name, synonym	Disease
application_id, funding_year, project_abstract, project_title, project_term, project_num, total_cost	Funded project
authors, (author) affiliation, (author) country, pmc number, pmid, pub_date, pub_issue, pub_page, pub_title, pub_volum, pub_year	Publication
org (organization), org_state (organization state), pi_id, pi_name	Principle investigator
agent name	Funding agent
journal_title	Journal

^a^MONDO ID was used as category_id because MONDO disease categories were adopted

### NIH funding knowledge graph

Based on the data model we described above, we loaded the mapped funding data to a knowledge graph hosted in Neo4j. To be specific, different types of data have been loaded and represented with those seven primary classes as nodes accordingly; object properties were applied to establish semantic connections between different nodes as edges, and data properties were attached to corresponding nodes as node properties. The knowledge graph is publicly assessable *without login requirement* at http://grants4rd.ncats.io:7474/browser/.

## Results

### Results of rare disease data preparation

A total of 5001 unique GARD rare diseases were categorized based on the MONDO disease classes. Table 3 shows the categorization results. Only 799 GARD diseases belonged to a single MONDO category, while most GARD diseases were mapped to multiple MONDO disease categories. For example, GARD:0006735 (Hypophosphatemic rickets) is mapped to three different MONDO disease categories: MONDO:0003847 (Mendelian disease), MONDO:0003900 (connective tissue disease), and MONDO:0021199 (disease by anatomical system). There are 52 GARD diseases that were not grouped into any of MONDO disease categories, because they were either mapped to obsolete MONDO diseases or another two MONDO disease category branches, “Disease Characteristic” and “Disease Susceptibility”, which were excluded from this study.

**Table 3:** Results of GARD diseases to MONDO disease categories

# GARD diseases	# MONDO disease categories
52	0
799	1
761	2
1156	3
645	4
540	5
504	6
332	7
133	8
49	9
28	10
2	11

### Results of NIH funding data mapping

#### Results of NIH funding data retrieval

We downloaded the funding data with funding year spanning from 1985 to 2019 from NIH ExPORTER [18]. A total of 2,457,303 distinct applications with 654,347 unique project titles, 886,895 unique project abstracts, and 2,555,300 publications are cleaned and stored in a MySQL database.

#### Results of NIH funding data mapped to GARD

Two types of mapping results have been generated via exact name (i.e., String) match and MetaMap annotations.

##### Disease name mapping results

Both GARD disease names and synonyms have been applied to map project titles. 1104 GARD diseases were mapped to 21,027 project titles, which correspond to 63,692 NIH funded applications. Since one project can be funded for multiple years as multiple applications with the same project title, the number of mapped applications is larger than the number of project titles.

##### MetaMap annotation results

652,975 unique project titles were annotated by the MetaMap annotator and 5,039,735 annotations were generated. To map GARD diseases to those annotations based on UMLS mappings, we first retrieved UMLS mappings for GARD diseases from GARD and MONDO. Specifically, 3468 GARD diseases with UMLS mappings were extracted from GARD, and out of 15,629 MONDO concepts with UMLS mappings, 3980 MONDO entries were mapped to 4032 GARD diseases and corresponding UMLS mappings, which were assigned to those GARD diseases. Together, 1146 GARD diseases were mapped to 13,695 project titles via UMLS mappings.

By merging mapping results from the above two steps, 1294 GARD diseases were successfully mapped to 72,577 funded applications, which corresponds to 45,647 distinct projects.

### Statistical results of NIH rare disease funding data in the Neo4j knowledge graph

**Fig. 2:**
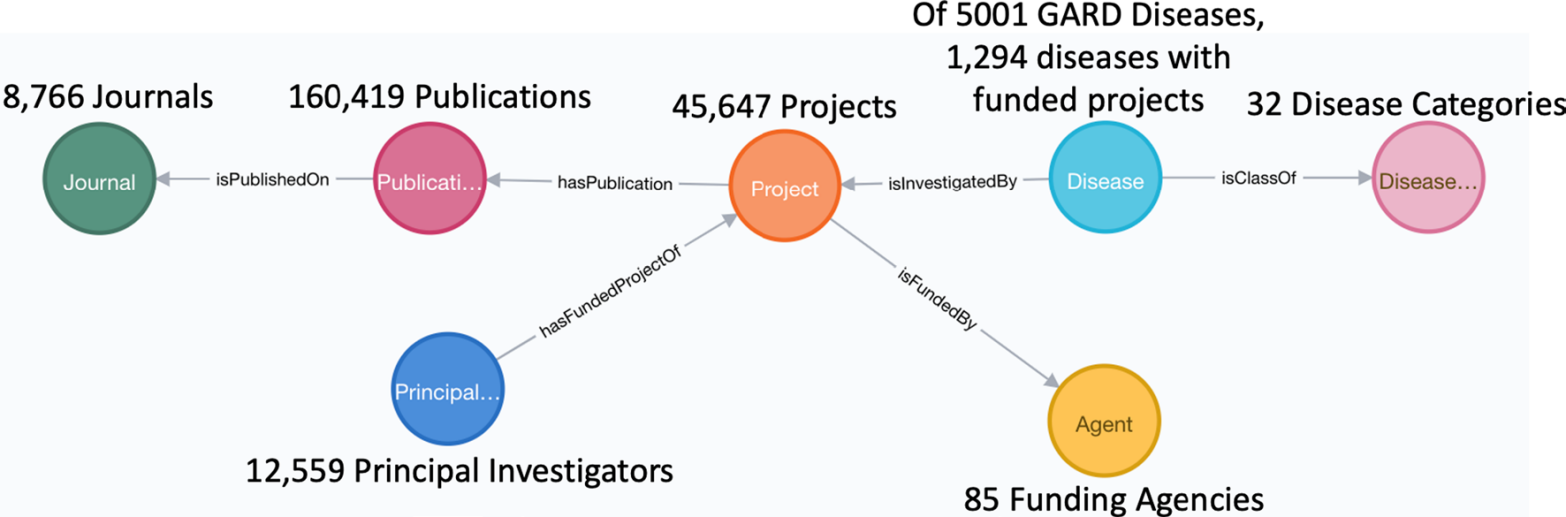
Statistics of rare disease funding Neo4j knowledge graph

Summarized statistical results of individual concepts belonging to each primary class are shown in Fig. 2.

## Case studies

To demonstrate the use of this integrated knowledge graph with rare disease funding data, we performed two types of case studies, (1) funding landscape assessments for an overview of the current funding landscape; (2) evidence-based research opportunity identification for supporting research in rare diseases. Cypher Queries have been composed and executed to perform case studies described in this section, and more details about those Cypher Queries are included in the Additional file 2.

### Current funding landscape

**Fig. 3:**
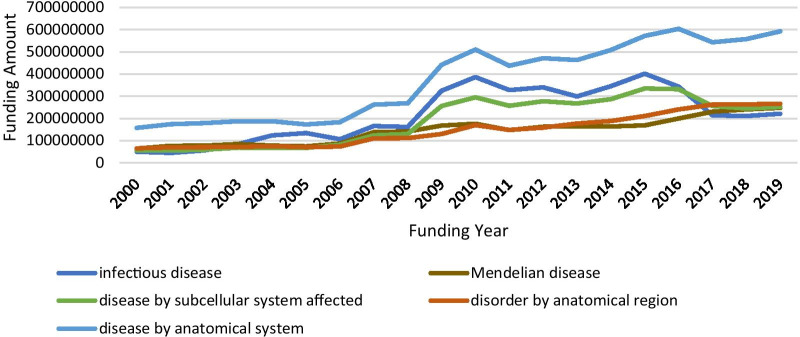
Funding trend by year for the top five most funded disease categories

To review NIH funding scenario in rare diseases, we composed several queries using Cypher, which is Neo4j’s graph query language that allows users to store and retrieve data from the graph database [21], to search against funding data in our Neo4j. Cypher Query 1 was constructed to access funding circumstances by disease categories and the result is listed in Table 4. Since one disease might be grouped into multiple disease categories, duplicates occurred when the number of GARD diseases, number of projects and total cost by each category were summed. Regardless, the numbers listed in Table 4 consistently reflect funding priorities with the consideration of disease burden applied by NIH. In addition, we retrieved funding amounts in the last twenty years for the top five most funded disease category, which is shown in Fig. 3. It is worthy to note that no funding cost data is available before the year of 2000 in our downloaded data. From Fig. 3, besides infectious diseases, big jumps were present due to the Ebola and Zika outbreaks that occurred during that time frame, the increasing funding trends are observed for other categories.

**Table 4:** Funding landscape in rare diseases by disease categories

Disease_Category	NumOfGARD	NumOfProjects	Total_Funding_Amount
Disease by anatomical system	982	34,373	7,470,493,496
Infectious disease	126	10,652	4,341,498,887
Disease by subcellular system affected	345	14,229	3,769,154,077
Disorder by anatomical region	475	15,261	2,934,525,892
Mendelian disease	504	15,244	2,892,410,817
Cell proliferation disorder	184	7964	1,619,194,341
Syndromic disease	269	5992	1,343,766,913
Inflammatory disease	101	2338	1,270,068,376
Acute disease	24	936	1,084,754,417
Disorder of development or morphogenesis	297	6053	1,050,936,925
Nutritional or metabolic disease	206	4896	930,754,532
Connective tissue disease	193	5684	913,060,844
Congenital abnormality	222	4391	847,471,304
Psychiatric disorder	101	2212	478,514,676
Systemic or rheumatic disease	67	1206	189,906,616
Post-infectious disorder	21	683	129,643,447
Pregnancy disorder	8	43	4,484,043
Disorder involving pain	2	23	4,140,753
Radiation or chemically induced disorder	6	72	3,140,791

#### Cypher Query 1

MATCH p = (n:Project)<-[:isInvestigatedBy]-(d:Disease)-[:isClassOf]-(c:DiseaseCategory) RETURN c.name AS Disease_Category, COUNT(DISTINCT d.gard_id) AS NumOfGARD, COUNT(n.application_id) AS NumOfProjects, SUM(TOINTEGER(n.total_cost)) AS Total_Funding_Amount ORDER BY Total_Funding_Amount DESC

We also evaluated the top 10 funded individual diseases (via Cypher Query 2). The top four funded diseases shown in Table 5 are infectious diseases. Poliomyelitis is considered a global public health emergency and the U.S. is a partner of the Global Polio Eradication Initiative [22]. Particularly, the CDC and the U.S. Agency for International Development (USAID) work to eradicate polio and have signed onto the Polio Endgame Strategy 2019–2023 [23]. About half of the world population is at risk for Malaria and the U.S. is the largest donor to the Global Fund to Fight AIDS, Tuberculosis, and Malaria (Global Fund) [24]. A large amount of research funds are being distributed on research for effective vaccines for Anthrax and Measles. In comparison to other rare diseases, the rest of diseases listed in Table 5, such as sickle cell disease with about 100,000 affected people in the US, both of cystic fibrosis and Huntington disease with more than 30,000 affected people in the US, are diseases that are closer to finding a cure or effective treatment, in part because they receive more research funding.

**Table 5:** Funding landscape in rare diseases by individual diseases

GARD_ID	GARD_Name	Total_Funding_Amount
GARD:0007413	Poliomyelitis	883,746,192
GARD:0006961	Malaria	767,571,217
GARD:0008157	Anthrax	586,088,491
GARD:0003434	Measles	506,370,796
GARD:0008614	Sickle cell anemia	409,771,041
GARD:0006233	Cystic fibrosis	379,448,373
GARD:0006677	Huntington disease	338,795,668
GARD:0007295	Ovarian cancer	329,709,694
GARD:0007108	Multiple myeloma	325,613,724
GARD:0009226	Abdominal obesity metabolic syndrome	306,250,394

#### Cyper Query 2

MATCH p = (d:Disease)-[:isInvestigatedBy]-(n:Project) RETURN d.gard_id AS GARD_ID, d.name AS GARD_Name, SUM(toInteger(n.total_cost)) AS Total_Funding_Amount ORDER BY total_cost DESC LIMIT 10

### Evidence-based rare disease research discovery

We aggregated funded projects and their publications into an integrated knowledge graph in Neo4j, which offers opportunities to programmatically support new research for rare diseases.

#### Research landscape assessment

Funded projects along with their publications show snapshots of their research goals and outcomes, which provides an opportunity to systematically assess the current research status and gaps, and consequently direct future areas for investigation. We enumerated two types of assessment for Neuronal ceroid lipofuscinosis (GARD: 0010739) and Duchenne Muscular Dystrophy (GARD:0007922) as examples.

##### Research status assessment for neuronal ceroid lipofuscinosis (NCL)

**Fig. 4:**
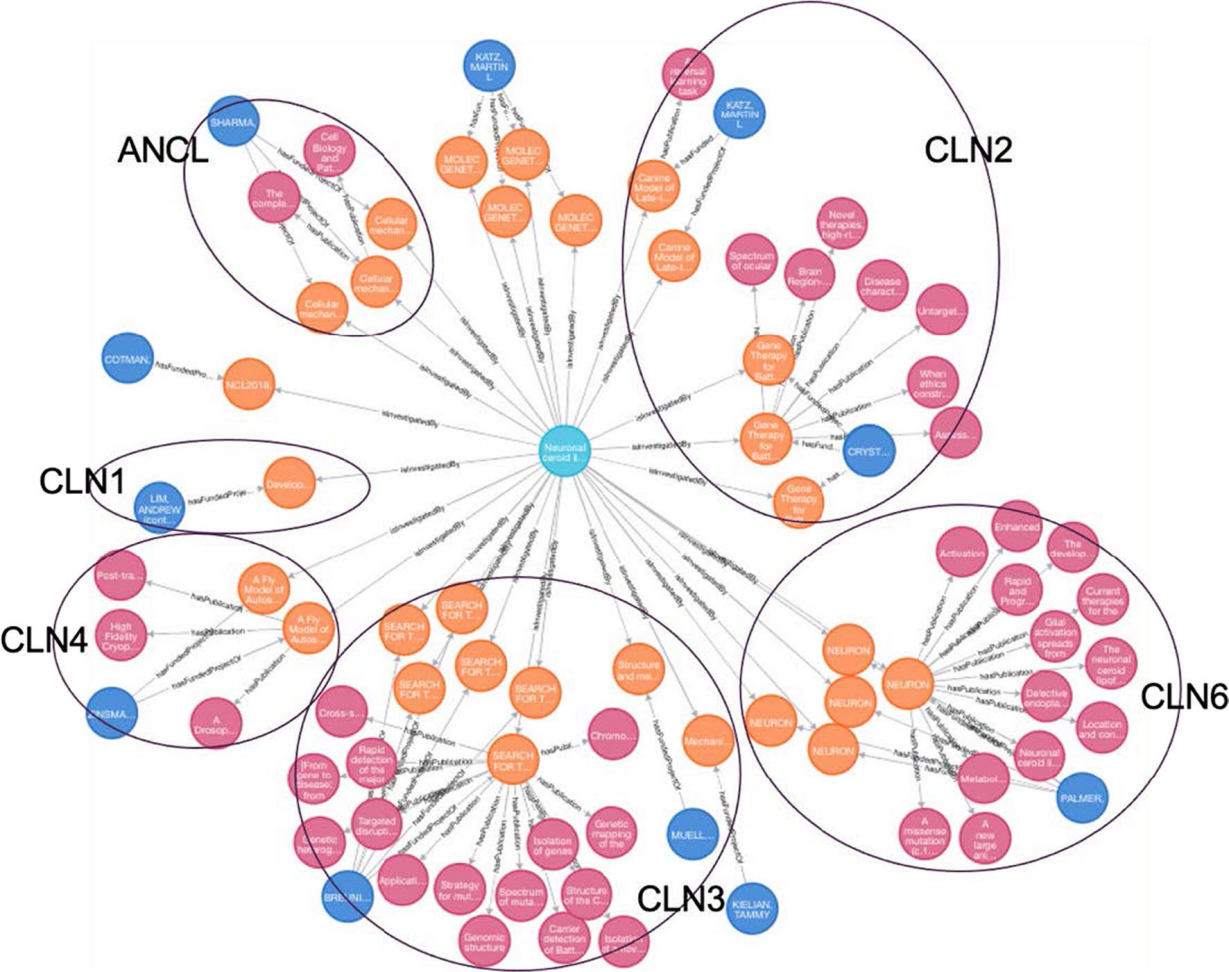
Research landscape of Neuronal ceroid lipofuscinosis (Light blue nodes denote diseases, orange nodes denote funded projects, magenta nodes denote publications, Dark blue nodes denote principal investigators. CLN1, CLN2, CLN3, CLN4, CLN6 and ACLN are different forms of NCL)

NCLs are classified by their causal gene of CLN (ceroid lipofuscinosis, neuronal), which is given a different number designation as its subtype. Signs and symptoms range in severity and progress at different rates given different gene mutations. The disorders generally include a combination of vision loss, epilepsy, and dementia. Some forms of the NCLs are: CLN1 disease, infantile onset; CLN2 disease, later-onset and so on [25]. Scientific investigations have been performed for each subtype of NCL (via Cypher Query 3). By manually examining the funded projects and their published studies for NCL, we grouped them based on their studied NCL forms (Fig. 4). Noticeably, these funded projects aim to better understand the molecular mechanism of NCL and discover therapeutic solutions. In particular, several subtypes (CLN2, CLN3 and CLN6) of NCL are more extensively studied than others. There is an Food and Drug Administration (FDA) approved enzyme replacement therapy for CLN2 disease (TTP1 deficiency) called cerliponase alfa (Brineura®) that has been shown to slow or halt the progression of symptoms [26]. With the exception of CLN2, which is highlighted as “CLN2” in Fig. 4, there are no approved treatments that can slow or stop disease progression for other forms of NCL disorder. As NCL affects the brain and nervous system, treatments must reach the brain to be effective, but getting the proper enzyme to cross the blood brain barrier can be difficult. For this reason, enzyme replacement therapy can only be used in NCL forms where the affected enzyme is soluble. This includes the subtypes known as CLN1, CLN2, CLN5 and CLN10 [27]. Thus, learning from the success of Brineura for CLN2 enhances the necessary scientific evidence and understanding of what existing research has been performed in other forms of NCL, and may better inform the direction of further research investigation on NCL.

#### Cypher Query 3

MATCH p = (d:Disease)-[:isInvestigatedBy]->(n:Project)<-[:hasFundedProjectOf]-(m:PrincipalInvestigator) WHERE d.gard_id = 'GARD:0010739' RETURN p

##### Research study clustering for Duchenne Muscular Dystrophy

**Fig. 5:**
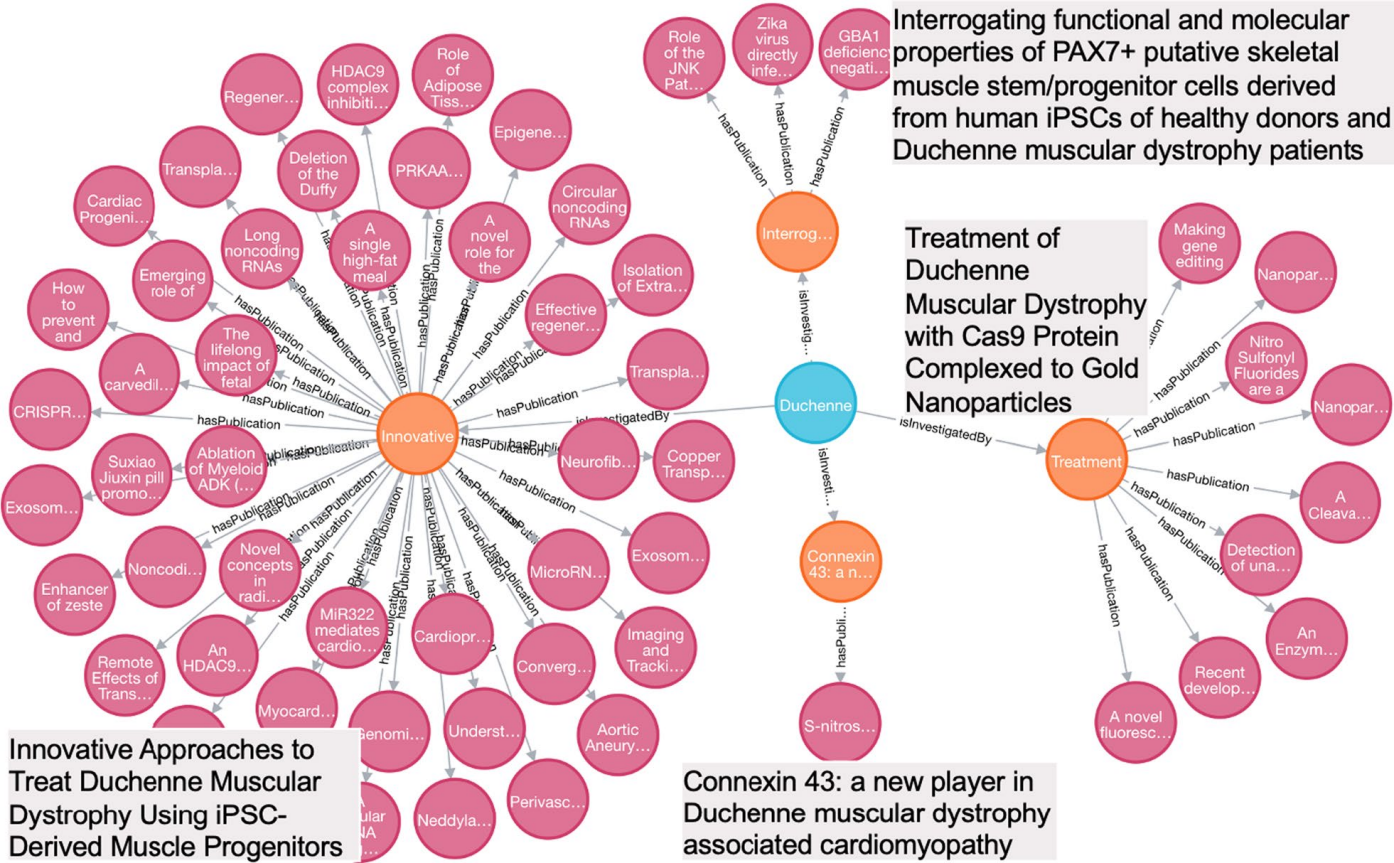
Publications clustered by funded projects for Duchenne Muscular Dystrophy (Blue nodes denote diseases, orange nodes denote funded projects, magenta nodes denote publications. Project titles are shown in the gray boxes)

Publications introduce novel approaches and/or findings that were proposed and generated from the funded projects. For example, a project entitled “LOCALIZATION OF X-LINKED HYPOPHOSPHATEMIC RICKETS GENE” (APPLICATION ID = “3087091”), has reported two publications, “Mutational analysis and genotype-phenotype correlation of the PHEX gene in X-linked hypophosphatemic rickets.” (PMID: 11502829) and “Mutational analysis of the PEX gene in patients with X-linked hypophosphatemic rickets.” (PMID: 9106524) These two publications specifically reported their investigation on genes for X-linked hypophosphatemic rickets. For a given disease, we clustered research studies by a project or a list of projects with similar research topics, which allows tracking research trajectory, identifying research gaps, and preparing necessary training data for future study. In this case study, we executed Cypher Query 4 to cluster publications associated with the funded projects for Duchenne Muscular Dystrophy (GARD:0006291), a lethal muscle wasting disease caused by the lack of dystrophin, which eventually leads to apoptosis of muscle cells and impaired muscle contractility. Four projects, along with their publications, are shown in Fig. 5. Based on the project titles shown in gray boxes in Fig. 5, we deduced that their objectives are all tied to breaching the major barriers to successful therapeutic solutions for Duchenne Muscular Dystrophy respectively via iPSCs, Cas9 and Cx43.

#### Cypher Query 4

MATCH p =(d:Disease)-[:isInvestigatedBy]-(n:Project)-[:hasPublication]-(m:Publication) WHERE d.gard_id = 'GARD:0006291' RETURN p

#### Identify new research opportunities

**Fig. 6:**
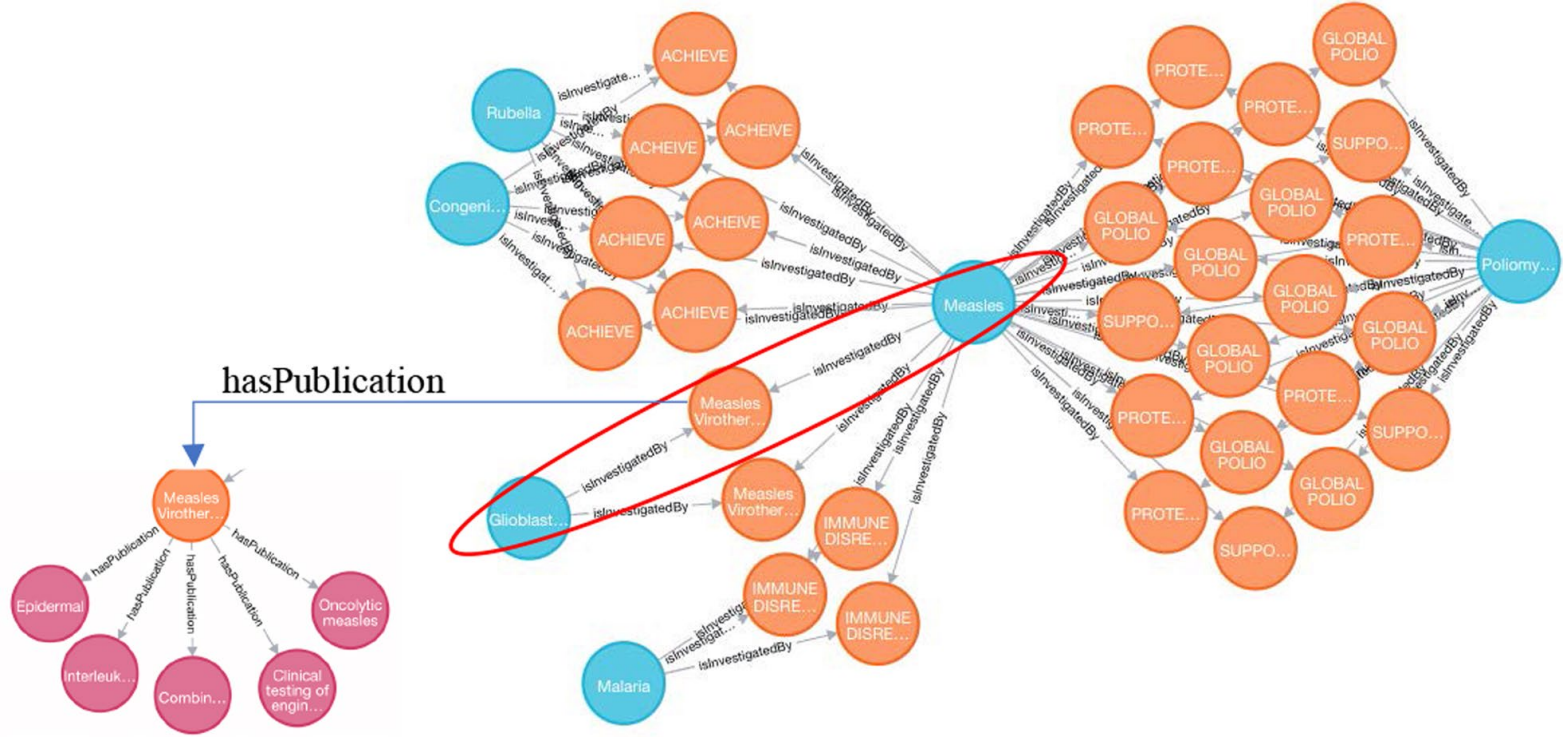
Identifying possible research opportunities for Measles via funded projects (Orange nodes denote funded projects, blue nodes denote rare diseases, magenta nodes denote publications)

In this case study, we proposed to gain research insights systematically by analyzing project titles. For instance, given one project entitled “USE OF LEUPROLIDE ACETATE FOR TREATMENT OF PRECOCIOUS PUBERTY”, we know this project was studying leuprolide acetate (Drug) for precocious puberty [28]. With the disease annotations generated for project titles (described in the “Identify new research opportunities” section), we were able to identify potential disease associations via projects, and furthermore to identify new avenues for research. Fig. 6 demonstrates an example of looking for potentially relevant diseases to Measles (GARD:0003434) via Cypher Query 5. Obviously multiple rare infectious diseases, including Poliomyelitis, Rubella, Congenital rubella, and Malaria are grouped together, and interestingly Measles and Glioblastoma is linked via one project entitled “Measles Virotherapy for Glioblastoma Multiforme”, highlighted in the red circle shown in Fig. 6. According to one recently published review paper written by Zhang et al. [29], “advances and potential pitfalls of oncolytic viruses expressing immunomodulatory transgene therapy for malignant gliomas”, the authors emphasized that the therapeutic efficacy of oncolytic viruses alone is limited, which might motivate novel research to better understand and assess the therapeutic efficacy. Five related publications produced by this project provide additional information to direct future investigation.

#### Cypher Query 5

MATCH p =(d:Disease)-[:isInvestigatedBy]-(n:Project)-[:isInvestigatedBy]-(d1:Disease) WHERE d.gard_id = 'GARD:0003434' RETURN p

## Discussion

In this study, we extracted NIH funding data for rare diseases and semantically represented it as a knowledge graph, which provides an effective way to not only assess the funding landscape in rare diseases, and systematically identify new research gaps/opportunities in rare diseases, but also share identified scientific findings/evidence with funding agencies for recommending their research fund distribution in rare diseases.

### Scientific observations from data preparation

Given various representations of disease names applied in the project titles, to accurately map diseases to projects via project titles and avoid any mis-mapping, we applied a complementary mapping strategy using string match and semantic annotation. For instance, one project entitled “Discovering Novel Treatments for Batten Disease” could only be mapped to “Batten Disease (GARD:0010739)” via string match based on the disease name, instead of semantic annotation. The MetaMap annotations generated from the project title is “Juvenile Neuronal Ceroid Lipofuscinosis (UMLS: C0751383)”, which is different from the UMLS mapping (UMLS:C0027877) associated with this GARD disease. Another project entitled “TOXIC METALS, MEMBRANE SIGNALING, AND CELL GROWTH” is mapped to “Heavy metal poisoning (GARD:0006577)” via semantic annotation instead of string match, since the MetaMap annotator generates one annotation of “toxic metals (UMLS: C0274869)”, which is mapped to the UMLS associated with that GARD disease. Clearly, even though we applied this complementary strategy to establish mappings between projects and diseases, we may miss relevant projects with the disease mentioned in the project abstract, instead of in the project title. To avoid any missing data, several additional steps have been proposed and described in the “Future work” section.

### Scientific insights derived from the case studies

We performed two types of case studies, funding landscape and research landscape analysis respectively to demonstrate the use of our funding knowledge graph for evidence-based rare disease research discovery.

#### Funding landscape assessment

Accessing funding data allows researchers to examine funding situations and funding opportunities in rare diseases. Our case studies illustrate that the funding trend consistently reflects the disease burden to the public health, e.g., infectious diseases received the most amount of funding support over years. A big jump regarding the total funding amount for infectious diseases between 2013 and 2017, coincides with the Ebola and Zika outbreaks that occurred during that timeframe. Another inspiring message derived from our analysis is that the amount of funding for supporting rare disease research has been increasing yearly, which might encourage and motivate more scientists to devote more of their research to rare diseases.

#### Scientific evidence-based rare disease research discovery

Analysis of funding data not only provides a systematic way to outline a complete research spectrum for an interested disease (category), but also generates scientific evidence supporting rare disease research discovery. For instance, the case study of Neuronal Ceroid Lipofuscinosis (NCL) illustrates that several different types of NCL have been investigated intensively and one therapeutic success on one type of NCL, namely, CLN2, which will enable us to dive into it and derive insight and knowledge about the status and gaps of research on NCL in further investigation. In addition, we demonstrated the power of aligning funded projects with their publications to direct research. As shown in the case study of Duchenne Muscular Dystrophy, it becomes feasible to cluster research papers based on a funded project or a list of projects with a similar research topic, which enables researchers to systematically track research pathway and programmatically prepare training data for future study. In this study, publication clusters were solely based on single projects; we propose to cluster publications with multiple projects with similar research topics by implementing Natural Language Processing (NLP) algorithms to analyze project abstracts.

### Limitations and future work

By reviewing the MONDO categories applied to organize rare diseases, some categories, for instance, “Mendelian disease”, “disease by anatomical system”, are broad categories consisted of many individual diseases; In addition, as shown in Table 3, most rare diseases are grouped into multiple MONDO categories. We propose to extend our disease category mapping to a higher granularity level as the next step, to precisely reflect funding distribution and assess research landscape by disease categories.

In this study, mappings between rare diseases and funded projects were established based on project titles. To avoid any mis-mappings with rare diseases that are mentioned in the project abstracts instead of project titles, we propose to expand our analysis with project abstracts. In addition, analyzing funding data from other resources, such as “funding” or “Acknowledgements” sections included in the publications to introduce the funding sources supporting their research, is another proposed extension task. Although the aforementioned extensions have been proposed as future work by aligning with our current project plan, we anticipated that we will still miss some data, such as, (1) projects without project titles and/or abstracts, (2) non-US government funded projects; (3) projects with only a single funding source (not all) was acknowledged in the publications. These are beyond the scope of this project and will be planned in the future study.

Insights and lessons drawn from the case studies were heavily relied on manual interpretation in this preliminary study, and we propose to transform currently manual processes into automated processes by implementing NLP and advanced machine learning techniques. Given the nature of rare diseases, limited data and knowledge about those diseases compared to common diseases, collaborative efforts seem more important and critical. We propose to discover methods to better promote research collaboration by connecting investigators with the requisite expertise and shared research interest from our funding knowledge graph. To that end, scientists can work collaboratively to pool patients, data, experience and resources together to support more innovative research in rare diseases. We are also interested in investigating the relationship between medical cost using information from datasets such as the AHRQ’s Healthcare Cost and Utilization Project (HCUP) [30] and research cost (i.e., NIH funding) in rare diseases, with the hypothesis that higher research funding should lead to improvements in earlier diagnosis of a rare disease and lower medical costs. Identifying overlaps and discrepancies between research status and medical situation will guide both further investigation and better inform decision makers on how to stimulate and advance research across more rare diseases.

## Conclusion

In this preliminary study, we developed an integrative knowledge graph to semantically represent NIH research funding data for rare diseases and successfully demonstrated its use of directing and promoting scientific evidence based rare disease research discovery. With the success and lessons learned from this study, we propose multiple improvements/extensions as the next step, to fully utilize funding data to accelerate the pace of rare disease research.

## Supplementary Information

**Additional file 1**. 32 root disease categories from MONDO.

**Additional file 2**. Cypher queries from the case studies.

## Data Availability

The data applied in this study can be accessed at http://grants4rd.ncats.io:7474/browser/.

## Abbreviations

GARD: Genetic and Rare Disease Information Center
MONDO: Mondo Disease Ontology
NGKG: NCATS GARD Knowledge Graph
UMLS: Unified Medical Language System
OBO: Open Biological and Biomedical Ontologies
NLP: Natural Language Processing
