# Supplementary material for: Scientific evidence based rare disease research discovery with research funding data in knowledge graph
Source: Orphanet J Rare Dis. 2021 Nov 18;16:483. doi: 10.1186/s13023-021-02120-9 (PMC8600882; doi:10.1186/s13023-021-02120-9)
Supplement: Supplementary file 2 — Additional file 2. Cypher queries from the case studies. [file 13023_2021_2120_MOESM2_ESM.docx]

| Case Study | Cypher Query Number | Cypher Query | Query Description |
| --- | --- | --- | --- |
| 1 | 1 | MATCH p = (n:Project)<-[:isInvestigatedBy]-(d:Disease)-[:isClassOf]-(c:DiseaseCategory) RETURN c.name AS Disease_Category, COUNT(DISTINCT d.gard_id) AS NumOfGARD, COUNT(n.application_id) AS NumOfProjects, SUM(TOINTEGER(n.total_cost)) AS Total_Funding_Amount ORDER BY Total_Funding_Amount DESC | To review funding distribution by disease categories, we searched for number of unique GARD diseases, number of funded projects and total funding amount by disease categories. |
| 1 | 2 | MATCH p = (d:Disease)-[:isInvestigatedBy]-(n:Project) RETURN d.gard_id AS GARD_ID, d.name AS GARD_Name, SUM(toInteger(n.total_cost)) AS Total_Funding_Amount ORDER BY total_cost DESC LIMIT 10 | To review the total funding amount for the top 10 most funded projects, we searched GARD ID, GARD name and total amount accordingly. |
| 2.1 | 3 | MATCH p = (d:Disease)-[:isInvestigatedBy]->(n:Project)<-[:hasFundedProjectOf]-(m:PrincipalInvestigator) WHERE d.gard_id = 'GARD:0010739' RETURN p | To review funding situation for Neuronal ceroid lipofuscinosis (NCL), we searched for all funded projects along with their principal investigators for NCL. |
| 2.1 | 4 | MATCH p =(d:Disease)-[:isInvestigatedBy]-(n:Project)-[:hasPublication]-(m:Publication) WHERE d.gard_id = 'GARD:0006291' RETURN p | To review research findings from funded projects for Duchenne Muscular Dystrophy, we searched for all funded projects and publications. |
| 2.2 | 5 | MATCH p =(d:Disease)-[:isInvestigatedBy]-(n:Project)-[:isInvestigatedBy]-(d1:Disease) WHERE d.gard_id = 'GARD:0003434' RETURN p | To identify potentially relevant diseases to Measles, we searched for diseases have been investigated by the funded projects, which were also targeted on investigation on Measles. |
